# The Matrisome as an Immunomodulator: A Role Far Beyond Its Structural Support

**DOI:** 10.3390/biom16030408

**Published:** 2026-03-10

**Authors:** Taihao Quan

**Affiliations:** Department of Dermatology, University of Michigan Medical School, Ann Arbor, MI 48109, USA; thquan@umich.edu; Tel.: +1-(734)-615-2403; Fax: +1-(734)-647-0076

**Keywords:** matrisome, matrikines, inflammation

## Abstract

The extracellular matrix (ECM) is a dynamic and complex three-dimensional network that provides structural support and mechanical stability to tissues. The complete repertoire of ECM and associated proteins has recently been cataloged as matrisome, which encompasses both core structural components and ECM-associated proteins. Advances in ECM biology have overturned the traditional view of the ECM as a purely passive scaffold, revealing its active involvement in a wide range of biological processes. Among these, the ECM plays a critical regulatory role in inflammation. This review examines the bidirectional interplay between the matrisome and inflammatory processes, highlighting how matrisome components shape inflammatory responses and how inflammation, in turn, drives matrisome remodeling. A deeper understanding of matrisome–inflammation interactions will provide important insights into immunopathology and may inform the development of novel therapeutic strategies.

## 1. Introduction

The extracellular matrix (ECM) comprises the most abundant proteins in our body (one-third of our body mass), and its dysregulation contributes significantly to most chronic diseases [1,2]. Fibrosis exemplifies this phenomenon, accounting for nearly half of all mortality cases and representing abnormal matrix behavior and inflammation [3]. Recently, the diverse components of the ECM have been collectively cataloged as the matrisome, which is largely produced by stromal cells [4,5]. The matrisome comprises over 1000 proteins and is organized into two main categories: the core matrisome and matrisome-associated proteins [6]. The core matrisome (e.g., Collagen I, Collagen IV, Collagen VI), ECM glycoproteins (e.g., fibronectin, laminin, tenascin-C), and proteoglycans (e.g., aggrecan, versican, perlecan), while the matrisome-associated proteins consist of ECM-affiliated proteins (e.g., aggrecan, versican, perlecan), ECM regulators (e.g., matrix metalloproteinases (MMPs), tissue inhibitors of metalloproteinases (TIMPs), and lysyl oxidase (LOX)), and secreted factors (e.g., transforming growth factor-β (TGF-β), Wnt ligands, and connective tissue growth factor (CTGF/CCN2)). Fibroblasts, which are the predominant stromal cells in the tissue, play an essential role in synthesizing these matrisome components and have long been recognized for providing structural support to the tissue [7,8].

The traditional view of ECM as passive structural proteins has undergone a paradigm shift in recent years [1,2,9,10]. Immune cells attach to and inhabit the ECM within tissue environments [1,11], yet not much research investigates the connections between ECM and immune cells. Despite the ECM making up roughly one-third of tissue composition, immunologists have largely overlooked its significance. Similarly, those studying matrix biology typically ignore how immune systems regulate these sophisticated structural networks. Investigating how stromal matrix properties and physical characteristics, including rigidity and mechanical forces, shape immune response represents a compelling research frontier.

Accumulating evidence demonstrates that matrisome are active participants in immune surveillance and inflammation, possessing the capacity to trigger danger signals, regulate inflammatory mediators, recruit immune cells, and modulate immune responses [1,10,12,13]. These immunomodulatory functions position matrisome as critical orchestrators of inflammation, influencing the initiation, progression, and resolution of inflammatory diseases.

Human skin is the most voluminous matrisome-rich tissue in the human body. The bulk of human skin is composed of dense collagen-rich matrisome, which is essential for the maintenance of skin structure, mechanical properties, and function [14,15,16]. The collagen-rich matrisome provides structural and mechanical support for skin while also modulating inflammatory responses [17,18,19]. Alterations of matrisome creates a tissue microenvironment with many pathologic skin disorders, such as increased fragility [20], impaired vasculature support [21], poor wound healing [22,23], skin dermal aging [14,15], and cancer development [14,15,24].

This review highlights current knowledge of the immunomodulatory roles of the matrisome in both innate and adaptive immunity, recognizing that these two arms of the immune response are substantially shaped by the matrisome microenvironment. This review examines how matrisome composition and remodeling influence immune cell behavior, and how the ECM acts as an active regulator of immune responses rather than a passive structural scaffold. Conversely, this review considers how immune activity itself reshapes the matrisome, establishing a bidirectional relationship that is central to both homeostasis and disease. Furthermore, this review explores the therapeutic implications of these findings and discusses future research directions that could translate insights from matrisome biology into novel treatment strategies for inflammatory conditions.

## 2. ECM and Immune Cell Bidirectional Interactions

**Figure 1:**
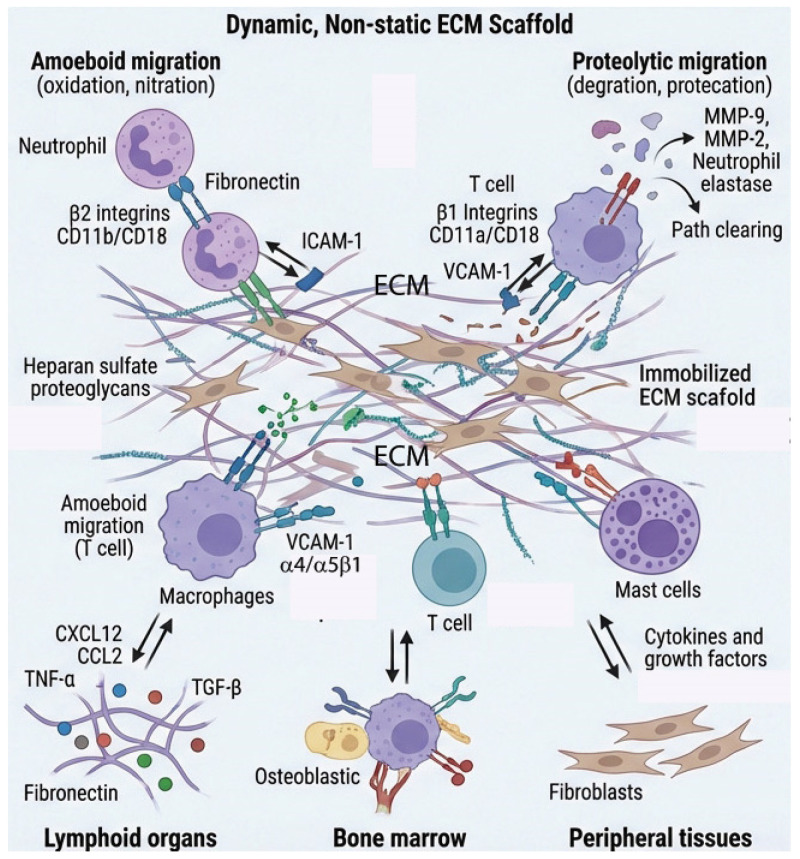
Bidirectional crosstalk between the ECM and immune cells. The ECM and immune cells engage in dynamic, reciprocal signaling that shapes tissue function in health and disease. ECM components, including structural proteins, proteoglycans, and matricellular proteins, regulate immune cell recruitment, activation, and effector functions through biochemical and mechanical cues. Conversely, immune cells secrete cytokines, chemokines, and matrix-remodeling enzymes that alter ECM composition, organization, and stiffness. These bidirectional interactions govern immune surveillance, modulate inflammatory responses, and influence outcomes in tissue repair and pathological conditions.

The ECM is not merely a static scaffold but rather a dynamic structure that profoundly influences immune cell behavior and inflammatory processes [1,2,25]. Recently, the relationship between ECM remodeling and inflammation is increasingly recognized as bidirectional and dynamic [1,11]. This reciprocal interaction creates a complex feedback loop where the physical and biochemical properties of the ECM influence inflammatory cell behavior and cytokine production, while simultaneously, inflammatory mediators drive ECM remodeling. The evolving paradigm highlights how tissue homeostasis, disease progression, and repair processes hinge on this molecular dialogue (Figure 1).

### 2.1. Integrin-Mediated Immune Cell–ECM Interactions

Integrins serve as the primary receptors mediating immune cell–ECM interactions [26,27]. These heterodimeric transmembrane receptors recognize specific ECM ligands and transduce bidirectional signals across the plasma membrane. The β2 integrin subfamily (CD18) is particularly important for leukocyte function [28]. It pairs with four different α subunits, αL (CD11a), αM (CD11b), αX (CD11c), and αD (CD11d), to form distinct receptors with specific ligand preferences. For instance, αMβ2 (Mac-1) expressed on neutrophils and macrophages binds to fibrinogen, iC3b, and denatured collagen, facilitating cell adhesion at inflammatory sites [29]. LFA-1 (αLβ2) primarily mediates leukocyte–endothelial and leukocyte–leukocyte interactions through ICAM-1 binding but also interacts with ECM components [30,31].

The β1 integrin family plays crucial roles in immune cell trafficking and retention [32]. α1β1 and α2β1 are collagen receptors that regulate T cell and dendritic cell positioning within tissues. α4β1 (VLA-4) binds to fibronectin and VCAM-1, enabling leukocyte rolling, firm adhesion, and transendothelial migration during recruitment to inflamed tissues [33]. α5β1 recognizes the RGD sequence in fibronectin and is important for T cell adhesion and migration [34].

This integrin-mediated crosstalk between immune cells and the ECM represents more than simple adhesion; it provides bidirectional signaling that integrates mechanical and biochemical cues from the tissue environment with intracellular signaling pathways. The specificity of integrin–ligand interactions, combined with their capacity for dynamic regulation through conformational changes and expression patterns, allows immune cells to navigate complex tissue architectures, respond appropriately to inflammatory signals, and execute localized effector functions. Understanding these integrin–ECM interactions is fundamental to comprehending immune cell biology.

The clinical success of natalizumab in multiple sclerosis (MC) and Crohn’s disease validates this mechanism, demonstrating that selectively inhibiting specific integrin pathways (α4β1/α4β7) can effectively reduce pathological immune trafficking while maintaining acceptable safety profiles [35,36,37]. Natalizumab prevents lymphocyte trafficking across the blood–brain barrier and gut endothelium by blocking α4β1/VCAM-1 and α4β7/MAdCAM-1 interactions. The later development of vedolizumab, a more selective α4β7 antagonist, established that refining the specificity of integrin blockade can yield a more favorable benefit–risk profile.

### 2.2. Immune Cell Migration Through ECM

The ECM creates specialized microenvironments or “niches” that support immune cell residence and function in specific tissues [38]. In lymphoid organs, the reticular network composed of collagen, fibronectin, and laminin provides structural support and creates distinct compartments for B and T cell zones [39]. Fibroblastic reticular cells produce ECM that organizes lymphocyte traffic and presents chemokines. In bone marrow, the ECM niche regulates hematopoietic stem cell maintenance and immune cell development [40,41]. Specific ECM components like osteopontin, tenascin-C, and various proteoglycans modulate stem cell quiescence versus proliferation [42,43]. In peripheral tissues, tissue-resident memory T cells and tissue-resident macrophages interact extensively with local ECM, which provides survival signals and maintains their phenotype [44].

Immune cells must navigate through complex three-dimensional ECM networks to reach sites of infection or injury [1]. This migration involves both proteolytic and non-proteolytic mechanisms [25]. Proteolytic migration requires matrix metalloprotease (MMPs) and other proteases secreted by migrating immune cells. Neutrophils release MMP-8 and MMP-9 to degrade collagen and gelatin, creating pathways through dense ECM [45,46]. Macrophages secrete a broad spectrum of MMPs including MMP-2, MMP-9, and MMP-12, enabling them to traverse basement membranes and interstitial matrices [47]. T cells upregulate MT1-MMP (MMP-14) upon activation, facilitating their penetration into inflamed tissues [48].

Non-proteolytic migration, or amoeboid movement, allows immune cells to squeeze through pre-existing pores in the ECM using cellular deformation and contractility [49,50]. This mode is particularly important for lymphocytes and is less dependent on integrin adhesion but requires coordination of actomyosin contractility and nuclear deformability. Amoeboid migration is ECM-dependent, as the pore size, stiffness, and composition of the matrix, including the abundance of hyaluronan and fibrillar collagens, directly influence the ability of cells to deform and translocate through the ECM.

### 2.3. ECM-Sequestered Factors and Immune Regulation

The ECM serves as a reservoir for cytokines, chemokines, and growth factors that regulate immune cell function [1,9]. These factors bind to ECM components through electrostatic interactions with glycosaminoglycans or through specific protein–protein interactions. Chemokines such as CCL2, CCL5, CXCL8, and CXCL12 bind to heparan sulfate proteoglycans, creating immobilized gradients that guide immune cell migration via “haptotaxis”, providing directional cues more stable than soluble gradients [51]. For instance, CXCL12 immobilized on bone marrow heparan sulfate retains lymphocytes in perivascular niches through CXCR4 signaling, while proteolytic release drives their rapid mobilization during inflammation [52,53]. CCL2 anchored to the endothelial glycocalyx promotes CCR2-dependent monocyte transendothelial migration, a process impaired when its glycosaminoglycan-binding residues are mutated [54,55]. Growth factors including TGF-β, VEGF, FGF, and PDGF are sequestered in the ECM in latent or bound forms and released upon ECM remodeling, influencing immune cell recruitment, differentiation, and resolution of inflammation [56]. Bioactive TGF-β, liberated by MMP cleavage or integrin αvβ6/αvβ8 engagement, suppresses dendritic cell maturation, promotes Treg differentiation, and drives M2 macrophage polarization; however, its dysregulated release in fibrotic or tumor microenvironments perpetuates immunosuppression and impairs effector T cell infiltration [57,58]. VEGF suppresses dendritic cell maturation, linking angiogenesis to local immune tolerance, while FGF-2 released from perlecan promotes mast cell survival and macrophage activation [59,60]. Importantly, the combinatorial and context-dependent presentation of ECM-sequestered factors, modulated by glycosaminoglycan sulfation patterns, ECM stiffness, and remodeling activity, fundamentally determines immune cell behavior. Rather than passive reservoirs, ECM-bound factors function as dynamic, spatially organized regulators whose bioavailability is tightly coupled to the structural and biochemical state of the surrounding matrix [11,61].

### 2.4. Immune Cell-Mediated ECM Remodeling

Immune cells actively remodel ECM, altering tissue architecture and creating a modified microenvironment that affects subsequent immune responses [62].

Neutrophils are early responders that release granule contents including neutrophil elastase, cathepsin G, proteinase-3, and MMPs, causing rapid ECM degradation [63]. They also release neutrophil extracellular traps (NETs) composed of DNA, histones, and proteases that can degrade ECM while trapping pathogens. Simultaneously, inflammation reduces the expression of TIMPs, further shifting the balance toward ECM degradation.

Macrophages exhibit phenotype-dependent ECM remodeling [62]. M1 macrophages secrete high levels of MMPs and produce ROS that damage ECM proteins, promoting degradation. M2 macrophages express lower MMP levels but produce TIMPs and secrete factors like TGF-β and PDGF that stimulate fibroblast ECM synthesis, supporting tissue repair and potentially fibrosis [64]. As such, while macrophages are well recognized for their role in ECM degradation, their contribution to ECM deposition is equally important [65,66]. Macrophages actively produce several ECM components, including fibronectin, select collagens, osteopontin, and matricellular proteins. This secretory capacity is particularly prominent in alternatively activated (M2-polarized) macrophages, which adopt a pro-fibrotic phenotype and drive fibroblast activation through TGF-β ligands secretion [62,67]. In chronic inflammatory conditions such as stromal fibrosis and atherosclerosis, sustained macrophage-driven ECM deposition can become pathological, reinforcing a pro-fibrotic matrisome that perpetuates tissue dysfunction. Recognizing macrophages as active contributors to both ECM synthesis and degradation therefore provides a more complete picture of how immune cells shape the matrisome microenvironment in health and disease. T cells influence ECM remodeling indirectly through cytokine production. Th1 cells secrete IFN-γ, which enhances macrophage MMP production, while Th2 cells produce IL-4 and IL-13, driving M2 macrophage polarization and fibrotic responses. Regulatory T cells (Tregs) can suppress excessive ECM degradation and promote resolution of inflammation [68].

Mast cells release tryptase and chymase, serine proteases that activate pro-MMPs and directly degrade ECM proteins [69]. They also secrete histamine and other mediators that increase vascular permeability, facilitating immune cell extravasation.

Inflammatory cells generate reactive oxygen species (ROS) and reactive nitrogen species (RNS) that chemically modify ECM proteins through oxidation, nitration, and glycation [70]. These modifications alter ECM mechanical properties, reduce its biological function, and create neoepitopes that can trigger further immune responses.

This multifaceted regulation of ECM remodeling by inflammation represents a double-edged sword: while acute inflammatory ECM remodeling facilitates tissue repair and pathogen clearance, chronic inflammation leads to pathological ECM changes that contribute to tissue dysfunction, fibrosis, and aging-related diseases. Thus, ECM remodeling is fundamentally intertwined with inflammation, orchestrating immune cell trafficking, activation, and function via direct receptor interactions, mechanical and biochemical signaling, and reciprocal modification by inflammatory mediators.

### 2.5. Pathological Immune Cell–ECM Interactions

Dysregulated immune cell–ECM interactions contribute to various pathological conditions [11,71]. In chronic inflammation, persistent immune cell infiltration and activation lead to excessive ECM degradation or pathological ECM accumulation (fibrosis) [72,73]. The altered ECM further perpetuates inflammation by presenting damage signals and maintaining pro-inflammatory immune cell phenotypes. In autoimmune diseases, immune cells may target ECM components directly (as in anti-collagen antibodies in rheumatoid arthritis) or indirectly damage ECM through chronic inflammation [74,75]. Modified ECM can then expose cryptic epitopes that become targets for autoimmune responses.

In cancer, the matrisome plays fundamentally different roles in “hot” (inflamed) versus “cold” (immune-excluded or immune-desert) tumors, primarily by shaping immune cell infiltration and function [76,77,78,79]. In hot tumors, the matrisome is relatively permissive to immune infiltration. ECM composition and remodeling enzymes do not form a dominant exclusionary barrier; instead, components such as certain laminins and fibronectin present chemokines and facilitate T cell trafficking, while pro-inflammatory damage-associated signals within the matrisome can further amplify immune activation [80,81]. In cold tumors, by contrast, the matrisome functions as an active barrier to immune infiltration. Dense collagen crosslinking, abundant fibronectin and tenascin-C deposition, and hyaluronan accumulation collectively produce a stiff, dense ECM shield that physically excludes T cells from the tumor core, trapping them in the stroma [82]. Beyond this mechanical exclusion, matrisome components in cold tumors actively suppress immune function through additional immunomodulatory mechanisms. The matrisome thus acts as a gatekeeper: open to immune engagement in hot tumors but remodeled into an immunosuppressive fortress in cold tumors. This distinction highlights ECM-targeting strategies as an important consideration for converting cold tumors into hot ones and, in turn, improving immunotherapy responses [83,84].

Inflammaging refers to chronic, low-grade, persistence inflammation that develops with advancing age, characterized by elevated levels of pro-inflammatory cytokines (such as IL-1β, IL-6, and TNF-α) in the absence of acute infection [85,86]. Inflammaging serves as a key driver of age-related diseases, arising from the cumulative activation of the innate immune system by cellular debris, senescent cells, and DAMPs that accumulate over a lifetime. In osteoarthritis (OA), for instance, progressive degradation of articular cartilage ECM, driven by elevated MMP-13 and ADAMTS-5 activity, releases fibronectin fragments and hyaluronan oligosaccharides that activate TLR-4 signaling in chondrocytes and synovial macrophages, perpetuating a self-reinforcing cycle of cartilage destruction and synovial inflammation [87,88]. As such, the ECM plays a significant role in this process, as age-related ECM damage and altered composition further perpetuate inflammaging; degraded ECM releases pro-inflammatory fragments, while loss of ECM mechanical integrity impairs immune cell migration and function [89]. The intricate interplay between immune cells and the ECM represents a fundamental aspect of tissue immunity and homeostasis. These bidirectional interactions regulate immune surveillance, control inflammatory responses, and determine outcomes in tissue repair and disease. Understanding these molecular dialogues offers promising avenues for therapeutic intervention in inflammatory diseases, fibrosis, cancer, and aging-related immune dysfunction.

## 3. Matrisome-Associated Proteins: MMPs as Inflammatory Modulators

Stromal fibroblasts are the primary regulators of ECM homeostasis, controlling both ECM synthesis and degradation. During inflammation, ECM remodeling becomes particularly active, with fibroblasts and immune cells modulating their production of MMPs and TIMPs in response to inflammatory cues, creating a complex regulatory network that governs tissue remodeling [90,91,92]. Both MMPs and TIMPs are matrisome components classified as ECM regulators within the ECM-associated proteins category.

MMPs constitute a family of zinc-dependent endopeptidases capable of degrading virtually all ECM components [93,94]. Skin dermal fibroblasts express numerous MMPs, including interstitial collagenases (MMP-1, MMP-8, MMP-13), gelatinases (MMP-2, MMP-9), stromelysins (MMP-3, MMP-10), membrane-type MMPs (MT-MMPs), and others [95]. Under homeostatic conditions, dermal fibroblasts maintain constitutive, low-level expression of most MMPs, ensuring balanced ECM turnover. However, inflammatory stimulation triggers dramatic shifts in MMP expression profiles, disrupting this equilibrium.

Pro-inflammatory cytokines, particularly IL-1β and TNF-α, are potent inducers of MMP expression in stromal fibroblasts [96,97]. These cytokines activate transcription factors, including activator protein-1 (AP-1) and nuclear factor-kappa B (NF-κB), which bind to promoter regions of MMP genes enhance transcription. The signaling cascades initiated by these cytokines engage mitogen-activated protein kinase (MAPK) pathways, which further amplify MMP expression through sustained transcriptional activation. IL-17, for instance, is increasingly recognized as a critical inflammatory mediator in chronic skin diseases such as psoriasis, and potently induces fibroblast MMP production, contributing to the persistent ECM remodeling characteristic of these conditions [98].

Beyond canonical role in ECM degradation, MMPs perform numerous regulatory functions that influence inflammatory processes. MMPs act as key modulators of the cytokine and chemokine network through selective proteolytic processing [99,100]. This process can either activate pro-forms of inflammatory mediators or inactivate them through strategic proteolytic truncation. Furthermore, MMPs liberate matrix-sequestered growth factors, including transforming growth factor-β (TGF-β) and vascular endothelial growth factor (VEGF), from their ECM reservoirs, making them bioavailable to surrounding cells and enabling context-dependent cellular responses [101]. This multifaceted activity positions MMPs as central regulators of both tissue remodeling and immune cell behavior during inflammatory responses.

During acute inflammation, increased MMP production by inflammatory cytokines facilitates immune cell migration by degrading ECM physical barriers [91,102]. This proteolytic remodeling creates permissive pathways that enable neutrophils, monocytes, and lymphocytes to efficiently navigate through tissue matrices to reach sites of infection or injury. The selective degradation of basement membrane components, particularly type IV collagen, laminin, and fibronectin, by specific MMPs (including MMP-2 and MMP-9) is critical for immune cell extravasation from the vasculature into inflamed tissues [103]. However, this proteolytic activity represents a double-edged sword in inflammatory responses. While controlled MMP activity supports beneficial immune surveillance and pathogen clearance, excessive or prolonged MMP activation can inflict substantial collateral tissue damage, degrade structural ECM components and disrupt tissue architecture. In chronic inflammation, persistent alterations in the MMP/TIMP balance contribute to pathological tissue remodeling [104,105]. Chronic overexpression of MMPs can lead to excessive ECM degradation, as observed in chronic wounds and some inflammatory diseases [106,107]. Conversely, chronically elevated TIMP levels or suppressed MMP expression contributes to fibrosis, as seen in systemic sclerosis and hypertrophic scarring.

**Figure 2:**
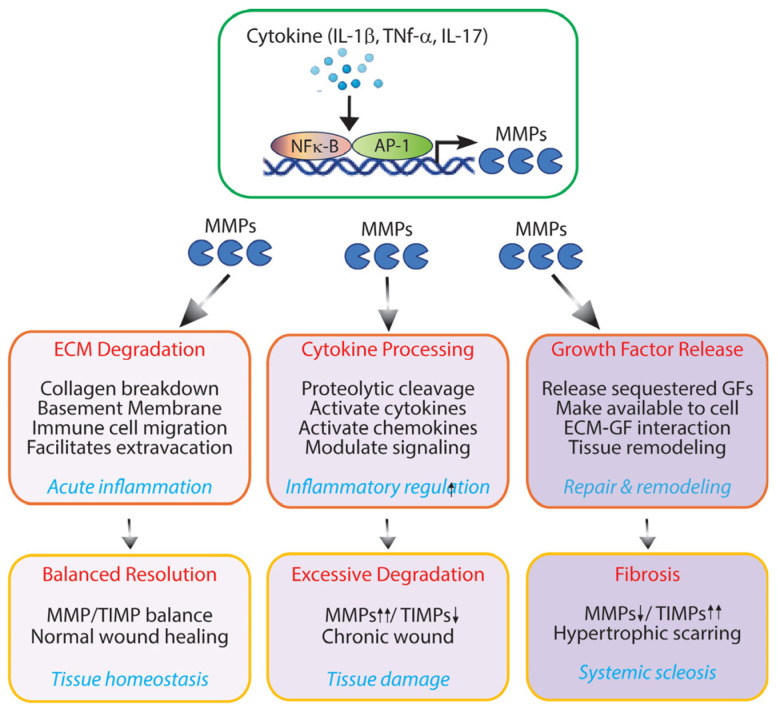
MMPs as mediators of inflammation. Pro-inflammatory cytokines (IL-1β, TNF-α, IL-17) activate transcription factors NFκ-B and AP-1 to upregulate MMP expression. Secreted MMPs execute three primary functions that determine tissue fate: (1) ECM degradation, breaking down collagen and basement membrane components to facilitate immune cell migration and extravasation during acute inflammation; (2) cytokine processing, proteolytically activating cytokines and chemokines while modulating inflammatory signaling cascades for inflammatory regulation; and (3) growth factor release, liberating sequestered growth factors from the ECM to enable cell–ECM-growth factor interactions critical for repair and remodeling. The balance between MMPs and TIMPs dictates three distinct pathological outcomes: balanced MMP/TIMP activity promotes normal wound healing and tissue homeostasis; excessive MMP activity with insufficient TIMP inhibition leads to pathologic ECM degradation, resulting in chronic non-healing wounds and tissue damage; conversely, reduced MMP activity with elevated TIMPs drives excessive matrix deposition, hypertrophic scarring, fibrosis, and systemic sclerosis.

Collectively, MMPs serve as pivotal mediators linking ECM remodeling to inflammatory processes in tissue (Figure 2). The balance between MMP activity and their endogenous inhibitors (TIMPs) critically determines whether acute inflammation is resolved with tissue restoration or progresses to chronic pathology characterized by persistent destruction and aberrant remodeling. Disruption of this balance, through sustained cytokine signaling, impaired TIMP production, or dysregulated MMP expression, perpetuates inflammatory cycles and compromises tissue integrity. This dual nature positions MMPs as both essential inflammatory mediators and promising therapeutic targets in inflammatory skin diseases, including psoriasis, atopic dermatitis, and chronic wounds.

## 4. Matrikines and Matrisome-Associated Proteins as Inflammatory Modulators

**Figure 3:**
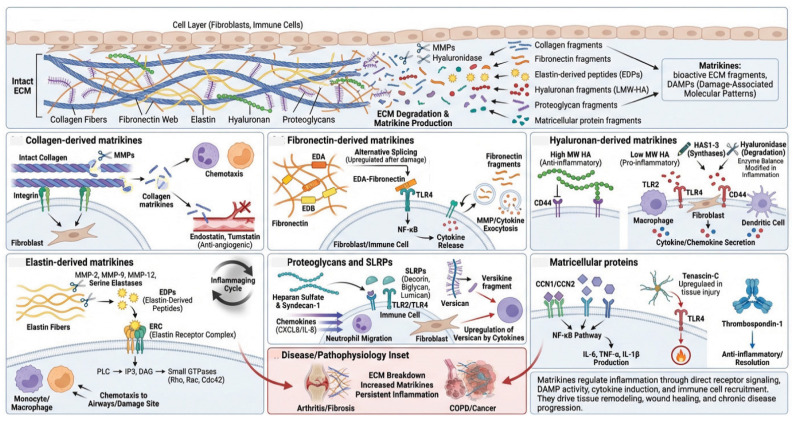
Matrikines regulate immune cell functions. Matrikines are bioactive ECM fragments generated by proteolytic cleavage of structural proteins including collagen, elastin, fibronectin, laminin, and hyaluronan by MMPs and other enzymes. These fragments bind specific receptors (integrins, TLRs, CD44) on immune cells such as neutrophils, macrophages, dendritic cells, and lymphocytes, activating signaling pathways (MAPK, NF-κB, PI3K/Akt) that regulate chemotaxis, cytokine production, and cellular activation. Matrikine–immune cell interactions modulate inflammatory responses and tissue remodeling outcomes, influencing the balance between acute inflammation, tissue repair, fibrosis, and restoration of homeostasis.

Matrikines are bioactive peptides and fragments liberated from ECM components upon proteolytic degradation [10,108,109]. Representative matrikines include collagen-derived peptides (e.g., endostatin from collagen XVIII, tumstatin from collagen IV), elastin-derived peptides (elastokines), laminin-derived fragments (e.g., laminin-111 peptides), fibronectin fragments (e.g., anastellin), and hyaluronan oligosaccharides. Unlike intact ECM, which primarily provides structural support and mediates cell attachment, matrikines possess distinct signaling capabilities as biologically active fragments. Matrikines exert their biological effects by interacting with specific cell surface receptors, including integrins, receptor tyrosine kinases, and Toll-like receptors, thereby modulating intracellular signaling pathways [108]. Additionally, matrikines function as DAMPs, propagating inflammatory signaling cascades [18]. Through these mechanisms, matrikines can influence diverse cellular behaviors, including proliferation, migration, differentiation, and inflammatory responses (Figure 3). Matrikines presence is especially significant in tissue remodeling, wound healing, and various pathological conditions where matrisome turnover is increased, such as arthritis, fibrosis, and cancer [2,110].

### 4.1. Collagen-Derived Matrikines

Collagens, the most abundant matrisome proteins, have diverse effects on inflammation beyond their structural roles [16,111]. Intact fibrillar collagens (types I and III) provide anchoring points for cells through collagen-binding integrin receptors, particularly integrin α11/β1 in skin dermal fibroblasts [112]. These integrin–collagen interactions deliver survival signals to fibroblasts and other cells and influence cell phenotypes. Collagen degradation products, however, can have pro-inflammatory effects [109]. Small collagen fragments act as chemotactic agents for fibroblasts and immune cells. Collagen fragments, particularly those derived from type I and type IV collagen, can act as chemoattractant for neutrophils and monocytes and stimulate angiogenesis [113]. Type IV collagen, a major component of basement membranes, is degraded during inflammation, facilitating immune cell migration [114,115]. Basement membrane remodeling is essential for angiogenesis during wound healing and chronic inflammation. Fragments of type IV collagen possess biological activities distinct from the intact molecule, including anti-angiogenic properties (as with endostatin and tumstatin, fragments derived from collagen XVIII and IV, respectively) [116].

### 4.2. Fibronectin-Derived Matrikines

Glycoproteins are a potentially important source of matrikines [10,110]. Fibronectin, a matrisome glycoprotein, plays multiple roles in inflammation [117,118]. Cellular fibronectin containing alternatively spliced domains (EDA and EDB) is upregulated during inflammation and wound healing [119]. EDA-fibronectin serves as an endogenous TLR4 ligand, activating fibroblasts and immune cells [117,118,120,121]. This creates an inflammatory positive feedback loop whereby tissue damage and inflammation induce production of EDA-fibronectin, which further activates inflammatory responses. Fibronectin also contains binding sites for various growth factors, chemokines, and other bioactive molecules, serving as a reservoir that concentrates these factors at appropriate tissue locations [122]. Fibronectin fragments generated during ECM degradation can have pro-inflammatory effects [123]. These fragments can activate fibroblasts through integrin signaling and potentially through TLRs, inducing MMP and cytokine production. The accumulation of fibronectin fragments in chronic wounds and inflammatory conditions may contribute to persistent inflammation [124,125].

### 4.3. Hyaluronan-Derived Matrikines

Hyaluronan (hyaluronic acid, HA) belongs to the proteoglycan category of the core matrisome. Hyaluronan, a glycosaminoglycan exhibits size-dependent effects on inflammation [126]. High molecular weight HA (>1000 kDa), predominant in healthy tissues, generally has anti-inflammatory and immunosuppressive properties. High MW HA signals through CD44 and other HA receptors to promote tissue integrity and inhibit inflammatory responses [127]. In contrast, low molecular weight HA fragments (<200 kDa), generated during tissue injury and inflammation, have pro-inflammatory effects [128]. HA fragments activate fibroblasts, macrophages, and dendritic cells through TLR2, TLR4 [129], and CD44 [130]. Upon binding to these receptors, HA fragments trigger downstream signaling cascades, most notably NF-κB and MAPK pathways, that drive the transcription of pro-inflammatory cytokines such as TNF-α, IL-1β, and IL-6, as well as chemokines that recruit additional immune cells to the site of injury [131]. CD44 engagement further promotes cytoskeletal reorganization and cell migration, facilitating fibroblast activation and myofibroblast differentiation, which can contribute to tissue remodeling and fibrosis in the context of chronic inflammation. Stromal fibroblasts both synthesize HA through hyaluronan synthases (HAS1, HAS2, HAS3) and degrade it through hyaluronidases [132,133]. During inflammation, stromal fibroblasts alter their HA synthesis patterns, sometimes producing more HA but also generating conditions conducive to HA fragmentation through increased hyaluronidase and reactive oxygen species [127]. The balance between intact and fragmented HA significantly influences the inflammatory environment.

### 4.4. Elastin-Derived Matrikines

Elastin is classified as an ECM glycoprotein within the core matrisome. Elastin is secreted as tropoelastin (the soluble precursor) and then cross-linked into insoluble elastic fibers that provide elasticity and resilience to tissues, such as arteries, lungs, and skin [134]. Elastin-derived peptides (EDPs), also known as elastokines, are bioactive fragments released during the proteolytic degradation of elastin [135]. EDPs are generated primarily through the action of MMPs, particularly MMP-2, MMP-9, and MMP-12, as well as serine elastases including neutrophil elastase and cathepsins [92,136]. EDPs exert their chemotactic effects primarily through interaction with the elastin receptor complex (ERC), which consists of three subunits: the peripheral 67 kDa elastin-binding protein (EBP), a catalytically inactive splice variant of β-galactosidase; the protective protein/cathepsin A (PPCA); and neuraminidase-1 (Neu-1) [137]. This receptor complex, expressed on immune cells including monocytes and macrophages, triggers a cascade of intracellular signaling events, including activation of phospholipase C and generation of inositol trisphosphate (IP3) and diacylglycerol (DAG), as well as activation of small GTPases (Rho, Rac, Cdc42) that drive actin polymerization and formation of lamellipodia and filopodia necessary for cell movement [138,139]. The chemotactic properties of EDPs create self-perpetuating inflammatory cycles in various pathological conditions: In COPD, cigarette smoke and inflammatory mediators stimulate elastase release, degrading lung elastin [140,141]. The resulting EDPs: Recruit additional inflammatory cells (neutrophils, macrophages, monocytes) to the airways. EDPs represent critical pathogenic mediators linking tissue degradation, inflammation, vascular disease, and aging [136]. Their ability to recruit immune cells creates self-amplifying cycles of matrix destruction and aberrant remodeling that characterize numerous chronic diseases. Recent evidence indicates that age-related elastin-derived fragments can evoke systemic inflammaging that drives aging processes [142,143]. Their ability to recruit immune cells creates self-amplifying cycles of matrix destruction and aberrant remodeling that characterize numerous chronic diseases. Targeting these molecules and their signaling pathways offers therapeutic strategies for conditions ranging from atherosclerosis to emphysema. Future research must focus on developing specific EDP antagonists, preventing elastin degradation, and promoting regenerative repair of elastic tissues to break the pathological cycles driven by these bioactive peptides.

### 4.5. Proteoglycans

Proteoglycans are one of the three core matrisome categories. Proteoglycans, consisting of core proteins with attached glycosaminoglycan chains, serve multiple functions in inflammation [144]. Proteoglycans bind chemokines to create haptotactic gradients that direct immune cell migration toward inflamed tissue niches [145]. For instance, heparan sulfate and syndecan-1 can sequester chemokines such as CXCL8 (IL-8), establishing gradients that enhance neutrophil infiltration [146]. Small leucine-rich proteoglycans (SLRPs) such as decorin, biglycan, and lumican are abundant in tissue stroma and can act as a DAMP, signaling through TLR2 and TLR4 to induce inflammatory responses [147,148]. Soluble biglycan, released during ECM degradation or actively secreted, activates immune cells and fibroblasts [149]. The pro-inflammatory effects of biglycan contribute to sterile inflammation, an inflammatory response initiated in the absence of pathogens, triggered by endogenous DAMPs including ECM fragments, oxidized lipids, uric acid crystals, and intracellular molecules released from damaged cells. This form of inflammation is particularly relevant in aging and chronic disease, where accumulated cellular damage perpetually activates innate immune pathways, contributing to the persistent low-grade inflammatory state characteristic of inflammaging. Lumican also has immunomodulatory properties, influencing immune cell migration and inflammatory responses [150].

Versican, a large chondroitin sulfate proteoglycan and member of the hyalectan family, is significantly upregulated in inflamed and remodeling tissues, where it plays multifaceted roles in tissue dynamics [151]. Stromal fibroblast production of versican is induced by inflammatory cytokines and growth factors, contributing to ECM changes during inflammation. Intact versican influences fundamental cellular behaviors including cell adhesion, migration, and proliferation by interacting with hyaluronan, cell surface receptors, and other ECM components. However, upon proteolytic cleavage by ADAMTS protease (metalloproteinase with thrombospondin motifs), versican generates bioactive fragments known as versikine, with exhibits distinct biological activities [152,153]. Versikine, particularly the G1 domain fragment, exhibits potent pro-inflammatory properties and can amplify inflammatory responses by engaging pattern recognition receptors such as TLR2 and activating NF-κB signaling pathways. Versikine also promotes leukocyte recruitment and cytokine production, creating a feed-forward inflammatory loop. Versican is robustly induced by inflammatory cytokines (including TNF-α and IL-1β) and growth factors (such as TGF-β and PDGF), contributing to the altered ECM composition and biomechanical properties characteristic of inflamed tissues [152,154]. This dynamic regulation of versican expression and processing represents a key mechanism by which the ECM actively participates in modulating inflammatory intensity and duration.

### 4.6. Matricellular Proteins

Matricellular proteins are a specific subset of matrisome-associated proteins that include: CCN family (CCN1/Cyr61, CCN2/CTGF, CCN3/NOV, CCN4, CCN5, CCN6), Thrombospondins (TSP-1, TSP-2), SPARC (secreted protein acidic and rich in cysteine), Tenascins (tenascin-C, tenascin-X), Osteopontin, and Periostin [155]. These proteins are secreted into the ECM but do not provide structural support. Instead, they modulate cell–matrix interactions and cell signaling, and regulate ECM assembly, remodeling, and cell behavior. Matricellular proteins are typically expressed at low levels in healthy adult tissues but are dramatically upregulated during development, wound healing, and inflammation.

CCN family proteins are a group of six secreted, matricellular proteins that play crucial roles in cellular communication and tissue regulation [156,157]. CCN proteins have emerged as important regulators of inflammatory responses, though their effects can be context dependent [155,158,159]. Several CCN proteins, particularly CCN1 and CCN2, can promote inflammation by stimulating the production of pro-inflammatory cytokines like IL-6, TNF-α, and IL-1β in immune cells and tissue-resident cells [160,161,162]. CCN1 can activate inflammatory signaling pathways including NF-κB, which is central to inflammatory gene expression. These proteins also promote leukocyte recruitment and adhesion to inflamed tissues [163]. They help coordinate the transition from acute inflammation to healing by promoting angiogenesis, fibroblast activation, and extracellular matrix remodeling. Dysregulated CCN protein expression is implicated in chronic inflammatory diseases including rheumatoid arthritis, atherosclerosis, inflammatory bowel disease, and fibrotic conditions [158,164]. Their dual nature makes them interesting therapeutic targets, though careful consideration of context is essential.

Tenascin-C is particularly noteworthy in the context of inflammation due to its dual structural and immunomodulatory functions [165]. This large hexameric glycoprotein is rapidly and dramatically upregulated in response to tissue injury and inflammation, with expression levels closely paralleling inflammatory intensity. Tenascin-C functions as an endogenous danger signal by directly activating TLR4 on immune cells and fibroblasts, thereby inducing pro-inflammatory responses including NF-κB activation and cytokine production [166,167]. Tenascin-C expression levels correlate with inflammatory activity and disease severity in conditions including rheumatoid arthritis, atherosclerosis, and chronic wounds, positioning it as both a biomarker and functional mediator of inflammation [168,169]. Additionally, tenascin-C modulates cell–ECM interactions through its ability to disrupt fibronectin-integrin binding, creating a “de-adhesive” microenvironment that promotes cell migration, a property critical for immune cell infiltration and fibroblast repositioning during tissue remodeling. Stromal fibroblasts are major producers of tenascin-C during inflammation, and its expression correlates with inflammatory activity in various diseases. Conversely, certain matricellular proteins such as thrombospondin-1 possess anti-inflammatory properties and promote resolution [170,171].

## 5. ECM Mechanical Properties as Inflammatory Modulators

**Figure 4:**
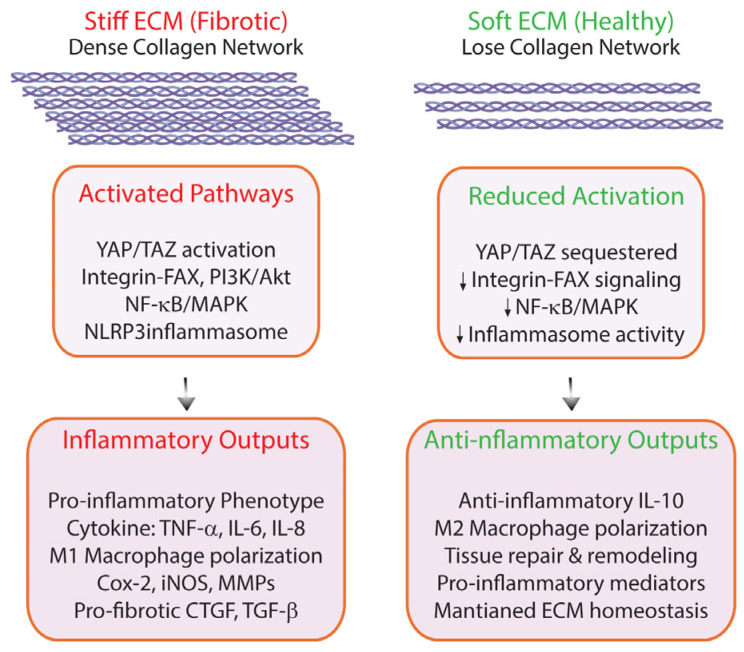
ECM mechanical properties as inflammatory modulators. ECM stiffness regulates inflammatory signaling through mechanotransduction. Stiff, fibrotic ECM (**left**) activates YAP/TAZ, integrin-FAX-PI3K/Akt, NF-κB/MAPK, and NLRP3 inflammasome pathways, driving pro-inflammatory cytokine production (TNF-α, IL-6, IL-8), M1 macrophage polarization, and pro-fibrotic factors (CTGF, TGF-β) that perpetuate inflammation and fibrosis. Soft, healthy ECM (**right**) sequesters YAP/TAZ and reduces pathway activation, promoting anti-inflammatory IL-10 production, M2 macrophage polarization, tissue repair, and ECM homeostasis. ECM mechanical properties thus serve as critical modulators of tissue inflammation in fibrotic diseases and conditions with abnormal mechanical loading.

Cells respond to both biochemical signals and mechanical cues within their environment [172,173]. The mechanical properties of the ECM, including its stiffness, elasticity, and topography, actively modulate immune cells through mechanical signals [174,175]. ECM mechanical properties can activate inflammatory signaling pathways, contributing to both the initiation and persistence of inflammation, particularly in conditions characterized by tissue fibrosis or abnormal mechanical loading [176,177] (Figure 4).

Matrix stiffness is determined by collagenous ECM content and crosslinking (via lysyl oxidase and AGEs), elastin fiber organization, proteoglycan hydration, and collagen alignment [178]. Physiological ranges span soft tissues (0.1–1 kPa), normal dermis (2–10 kPa), and fibrotic/aged skin (10–50+ kPa) [179]. Measurement of mechanical properties includes: atomic force microscopy (AFM) for nanoscale indentation mapping via cantilever deflection; rheometer for bulk viscoelastic moduli through oscillatory stress; and tension sensors for real-time force visualization using fluorescent/mechanical reporters. These techniques characterize mechanics from molecular to tissue scales.

Macrophages are especially sensitive to substrate rigidity, altering their gene expression profiles and polarization states: stiffer, fibrotic matrices promote the pro-inflammatory M1 phenotype, characterized by enhanced production of cytokines such as TNF-α and IL-6, whereas softer matrices favor the anti-inflammatory M2 phenotype, which secretes IL-10 and participates in tissue repair [180,181]. This mechanical sensitivity is also noted in other cell types such as dendritic cells, which modulate their activation in response to changes in ECM compliance [182,183]. Furthermore, mechanical cues can influence the threshold and duration of immune cell activation, serving as a “danger signal” in the context of tissue injury and fibrosis [184].

Mechanosensitive pathways, encompassing integrins, focal adhesion complexes, and transcriptional regulators, enable cells to detect changes in tissue stiffness, stretch, and deformation [185]. The Hippo pathway effectors Yes-associated protein (YAP) and transcriptional coactivator with PDZ-binding motif (TAZ) have emerged as critical mechanosensitive transcriptional regulators in cells [186]. Unlike traditional transcription factors, YAP/TAZ lack DNA-binding domains and instead function as transcriptional coactivators by partnering with TEAD family transcription factors to regulate genes involved in cell proliferation, survival, and ECM remodeling [187]. The subcellular localization of YAP/TAZ is exquisitely sensitive to mechanical cues: high mechanical tension promotes nuclear accumulation and transcriptional activity, while low tension or soft substrates result in cytoplasmic sequestration and degradation [188]. Nuclear YAP/TAZ enhances expression of pro-inflammatory cytokines including IL-6, IL-8, and CCL2, creating a mechanically driven inflammatory milieu [189]. Additionally, YAP/TAZ drive the expression of connective tissue growth factor (CCN2/CTGF), transforming growth factor-β (TGF-β), and matrix metalloproteinases (MMPs), establishing a pro-fibrotic program that sustains fibroblast activation and amplifies inflammatory signaling [190,191,192].

YAP/TAZ signaling in stromal fibroblasts directly upregulates numerous inflammatory mediators [193]. The age-related decline in YAP/TAZ activity, driven by mechanical deterioration of the dermal ECM, represents a key mechanism underlying inflammaging in skin aging [89]. Single-cell RNA-seq analysis revealed that decreased expression of the YAP/TAZ signature gene set represents a primary hallmark of aged mouse dermal fibroblasts, strongly supporting the role of ECM mechanical defects in regulating YAP/TAZ activity and inflammaging. Consistent with this, YAP/TAZ signaling is impaired in aged human skin, which exhibits characteristic inflammaging features [15,192]. This impairment results from age-related increases in collagen fragmentation, a prominent feature of dermal aging. This mechanical-inflammatory coupling creates a pathological feedforward loop in which initial mechanical stress triggers inflammatory responses that further alter tissue mechanics, thereby sustaining chronic inflammation and progressive tissue dysfunction.

Mechanical stress activates multiple inflammatory signaling pathways in dermal fibroblasts beyond YAP/TAZ nuclear translocation. Stretch-activated ion channels, particularly transient receptor potential (TRP) channels (TRPV4, TRPC1) and Piezo channels (Piezo1/2), serve as primary mechanosensors that respond to membrane deformation and cytoskeletal tension by permitting rapid calcium influx [194,195]. This calcium surge triggers downstream inflammatory cascades including NF-κB activation (promoting IL-6, IL-8, and TNF-α transcription) and MAPK pathway stimulation (ERK1/2, p38, JNK), which amplify pro-inflammatory gene expression.

Mechanical activation of integrin–FAK complexes stimulates phosphoinositide 3-kinase (PI3K)/Akt signaling, which cross-talks with inflammatory pathways to enhance production of prostaglandins, leukotrienes, and reactive oxygen species [196,197].

The inflammasome, particularly the NLRP3 complex, can be activated by mechanical stress in fibroblasts through mechanisms involving mitochondrial dysfunction, ATP release, and potassium efflux [198]. Mechanically stressed fibroblasts exhibit increased caspase-1 activation and secretion of mature IL-1β and IL-18, pro-inflammatory cytokine that amplify local inflammatory responses and recruit immune cells [199,200]. Furthermore, mechanical stress enhances expression and activity of cyclooxygenase-2 (COX-2) and inducible nitric oxide synthase (iNOS), generating inflammatory lipid mediators and reactive nitrogen species that contribute to tissue damage and sustained inflammation [201,202].

## 6. Matrisome-Related Senescence-Associated Secretory Phenotype (SASP) Factors as Inflammatory Modulators

The Senescence-Associated Secretory Phenotype (SASP) refers to the complex mixture of factors that senescent cells secrete into their surrounding environment [203]. When cells become senescent (they stop dividing but remain metabolically active), they become highly secretory, releasing SASP factors. Senescent fibroblasts accumulate in chronically inflamed and aged skin and exhibit a SASP characterized by persistent secretion of inflammatory cytokines, chemokines, and MMPs [204]. These senescent fibroblasts contribute to inflammaging, which represents a hallmark of aging [205]. The SASP comprises several matrisome components, including ECM regulators such as MMPs, particularly MMP-1, MMP-3, and MMP-9, which establish a pro-inflammatory microenvironment that influences ECM remodeling and sustains chronic inflammation. Furthermore, senescent fibroblasts exhibit impaired core matrisome synthesis with reduced production of type I and type III collagen, elastin, and proteoglycans, while simultaneously increasing ECM degradation, thereby driving progressive matrix deterioration including altered matrix mechanical properties [206].

**Figure 5:**
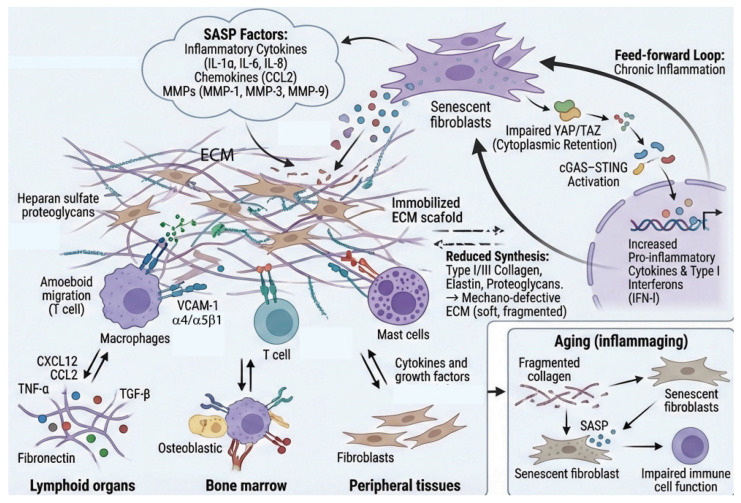
Matrisome-related SASP factors as inflammatory modulators. Senescent fibroblasts accumulate with age and secrete SASP factors, which drive inflammaging, a hallmark of aging. Key matrisome-related SASP components include ECM-degrading enzymes (MMP-1, MMP-3, MMP-9), ECM regulatory proteins (TIMPs, matricellular proteins), and pro-inflammatory mediators (IL-6, IL-8, IL-1, CCL2, TGF-β, VEGF). The diagram illustrates how senescent fibroblasts create a pro-inflammatory microenvironment that perpetuates ECM degradation, sustains chronic inflammation, and contributes to age-related tissue dysfunction, representing a potential therapeutic target for healthy aging interventions.

Collectively, these alterations in ECM composition and mechanical properties create a tissue microenvironment that modulates immune cell behavior, including recruitment, activation state, and functional responses, potentially perpetuating inflammatory signaling and impairing tissue repair. One convincing example is a recent report showing that senescence-associated mechano-defective dermal ECM impairs YAP/TAZ activity, which in turn unleashes cGAS–STING signaling and leads to skin dermal aging in mice [89]. YAP/TAZ activity is indeed impaired in aged human skin due to age-related decline in dermal ECM mechanical properties, contributing to skin aging through diminished collagenous ECM production [14,15,192,193,207]. This mechanosensitive signaling pathway, which responds to ECM stiffness and mechanical cues, becomes progressively dysregulated with advancing age as the dermis loses structural integrity and biomechanical properties. Specifically, when YAP/TAZ transcriptional activity is diminished due to mechano-defective dermal ECM, cytoplasmic DNA accumulates and activates the cyclic GMP-AMP synthase (cGAS), which then produces the second messenger cGAMP. This molecule binds to and activates STING (stimulator of interferon genes), triggering a cascade of inflammatory responses including the secretion of pro-inflammatory cytokines and type I interferons. This mechanically regulated cGAS–STING axis represents a critical link between mechano-defective ECM and the SASP that characterizes aged skin. The loss of YAP/TAZ-mediated mechanotransduction not only impairs fibroblast synthetic capacity for ECM components like collagen and elastin, but also creates a feed-forward loop wherein reduced ECM production further diminishes mechanical signaling, perpetuating inflammation and accelerating tissue aging. Understanding this mechanobiological mechanism has important implications for developing interventions that target ECM restoration, YAP/TAZ reactivation, or modulation of the cGAS–STING pathway to ameliorate age-related skin degeneration (Figure 5).

## 7. Matrisome-Targeted Therapeutic Implications

**Figure 6:**
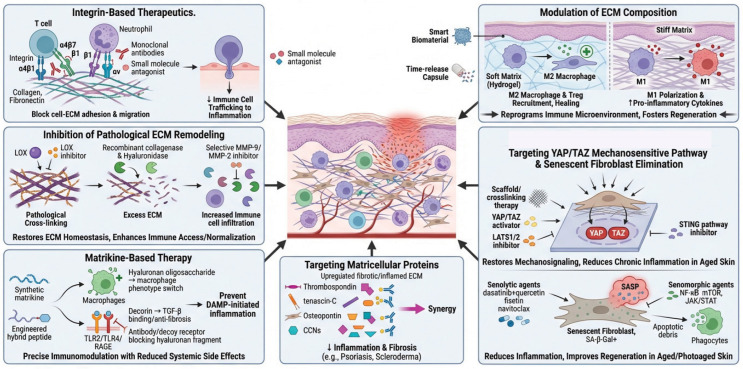
Therapeutic strategies targeting the immune–matrisome interface. The matrisome has emerged as a critical regulator of inflammation, shifting from a view of the ECM as a passive structural scaffold to an active participant in immune responses. This paradigm shift has enabled development of diverse therapeutic approaches that target immune cell–matrisome interactions: (1) direct inhibition of immune cell adhesion and migration through blockade of integrin–ECM or CD44–hyaluronan interactions; (2) modulation of ECM composition via MMP inhibitors or crosslinking enzyme antagonists; (3) manipulation of ECM mechanical properties to alter immune cell behavior; (4) targeting bioactive matrikines that propagate inflammatory signals; and (5) delivery of engineered biomaterials or decellularized matrices to redirect tissue remodeling. These matrisome-targeted interventions hold promise across autoimmune disorders, fibrotic diseases, cancer immunotherapy, and regenerative medicine applications where dysregulated immune–ECM crosstalk drives pathology.

The recognition of ECM components as active orchestrators rather than passive bystanders in inflammation has catalyzed the development of multiple therapeutic avenues targeting the immune-matrisome/ECM interface [1,109,208]. These strategies range from direct blockade of immune cell-matrisome/ECM interactions to sophisticated modulation of matrisome/ECM composition, mechanical properties, and matrikines (Figure 6).

### 7.1. Integrin-Based Therapeutics: Disrupting Immune Cell-ECM Adhesion

Integrin-blocking antibodies represent one of the most clinically advanced approaches for targeting immune cell–ECM interactions [209,210]. By preventing integrin-mediated adhesion and migration, these agents may effectively reduce immune cell trafficking into inflamed tissues. The therapeutic success of natalizumab across multiple sclerosis and Crohn’s disease confirms selective blockage of α4β1 and α4β7 integrin pathways can effectively reduce pathological immune trafficking while maintaining acceptable safety profiles [35,36,37]. In dermatology, efalizumab previously targeted αLβ2 integrin (LFA-1) to limit T-cell activation and migration in psoriasis, though it was subsequently withdrawn due to safety concerns regarding progressive multifocal leukoencephalopathy risk [211]. Small-molecule integrin antagonists and more selective antibodies targeting β1, β2, and αv integrins are currently in preclinical and clinical development for various inflammatory skin conditions [209,212].

Future therapeutic development should prioritize highly selective integrin antagonists to block pathological immune cell migration in specific tissues while minimizing systemic immunosuppression. ECM-based therapies like natalizumab (for MS and Crohn’s disease) effectively reduce immune cell infiltration but carry risks such as progressive multifocal leukoencephalopathy due to broad immunosuppression [213]. Vedolizumab offers gut-specific action with less systemic effect but is limited outside gastrointestinal disorders [214]. These findings highlight the need for tissue-specific approaches, temporary inhibition, or targeting integrins on pathogenic immune cells. Preclinical data suggest local modulation of ECM or integrin expression can improve precision, but off-target effects and disrupted tissue repair remain concerns. Pathology-specific strategies are crucial to maximize therapeutic benefit and limit risks.

### 7.2. Targeting ECM Composition to Modulate Immune Cell Behavior

Beyond direct pharmacological intervention, modulating ECM composition offers an indirect yet powerful strategy for controlling immune cell behavior [215,216]. In tissue engineering and regenerative medicine applications, biomaterials and scaffolds engineered to recapitulate native ECM characteristics can profoundly influence immune responses [217]. Matrix stiffness, topography, ligand density, and degradation kinetics collectively determine whether an implanted material promotes inflammatory or pro-regenerative immune polarization [218,219]. Soft, compliant hydrogels mimicking the mechanical properties of healthy dermis tend to favor M2 macrophage polarization and regulatory T-cell recruitment, whereas stiff matrices promote M1 activation and pro-inflammatory cytokine production [220,221,222]. These principles are being translated into therapeutic applications for chronic wounds, where bioengineered ECM scaffolds incorporating anti-inflammatory signals can redirect dysfunctional immune responses and promote healing [223,224]. While ECM-based strategies offer powerful tools for modulating immunity, their efficacy is context-dependent, and optimizing biomaterial properties for specific pathological environments is essential to avoid adverse outcomes.

Future therapeutic strategies should aim to develop “smart” biomaterials capable of sensing and adapting to the inflammatory microenvironment in real time, thereby actively directing immune cell behavior toward tissue repair rather than prolonged inflammation. Incorporating bioactive ECM fragments, growth factors, or immunomodulatory peptides with controlled release profiles can offer precise, phase-specific modulation of immune responses, delivering pro-regenerative cues exactly when needed to optimize healing.

### 7.3. Targeting Pathological ECM Remodeling and Mechanical Properties

Therapeutic strategies aimed at reversing or preventing pathological ECM remodeling are advancing on multiple fronts, with particular relevance for fibrotic skin diseases and tumor-associated inflammation [208,225]. Lysyl oxidase (LOX) and LOX-like enzymes catalyze collagen and elastin cross-linking, contributing to tissue fibrosis and creating a pro-tumorigenic microenvironment. Development of highly selective LOX inhibitors designed to disrupt pathological collagen cross-linking and fibrotic processes while preserving normal extracellular matrix remodeling and homeostatic tissue maintenance [226,227]. Delivery strategies ensuring tissue-specific or cell-type-specific targeting will be critical for clinical success. ECM-degrading enzymes, including recombinant collagenase and hyaluronidase, are being investigated for their ability to normalize fibrotic tissue mechanical properties and architecture as well as enhance immune cell infiltration in solid tumors [208,228,229]. By breaking down excessive or aberrantly cross-linked matrix, these enzymes can restore tissue compliance and improve accessibility for therapeutic immune cells in immunotherapy contexts. Small-molecule LOX inhibitors and neutralizing antibodies have shown promise in preclinical models of fibrotic diseases and cancer by reducing matrix stiffness, normalizing tissue mechanics, and improving immune cell infiltration [230,231,232].

MMP modulation presents a more nuanced therapeutic challenge, as these enzymes can both promote and resolve inflammation depending on context. While broad-spectrum MMP inhibitors have largely failed in clinical trials due to lack of specificity and disruption of homeostatic ECM turnover, selective targeting of specific MMPs implicated in pathological remodeling (such as MMP-9 in chronic wounds or MMP-2 in keloids) remains under investigation [233,234,235]. Tissue inhibitors of metalloproteinases (TIMPs) have also been explored as therapeutic agents, though their pleiotropic effects on multiple MMPs complicate clinical translation [236].

Future therapeutic strategies for pathological ECM remodeling should focus on developing highly selective LOX inhibitors with tissue-specific delivery systems to disrupt fibrotic cross-linking while preserving normal matrix homeostasis, particularly for fibrotic skin diseases and tumor microenvironments. Combining ECM-degrading enzymes (recombinant collagenase, hyaluronidase) with immunotherapy represents a promising approach to normalize tissue mechanics, reduce matrix stiffness, and enhance immune cell infiltration in solid tumors. Rather than broad-spectrum MMP inhibition, future efforts should target specific MMPs involved in pathological remodeling (MMP-9 in chronic wounds, MMP-2 in keloids) using context-dependent strategies that account for their dual pro-inflammatory and resolving roles. Success will require precision medicine approaches that consider disease stage, tissue context, and the balance between pathological remodeling and essential physiological ECM turnover, potentially utilizing combination therapies that coordinate matrix normalization with immune modulation or regenerative interventions.

### 7.4. Matricellular Proteins as Therapeutic Targets

Matricellular proteins, including thrombospondins, tenascins, osteopontin, and CCN family members, present promising therapeutic opportunities for inflammatory and fibrotic skin diseases due to their regulatory roles in immune cell recruitment, activation, and ECM deposition without providing structural support [155,237]. Thrombospondins, tenascins, osteopontin, and members of the CCN family are upregulated during skin inflammation and fibrosis, where they promote immune cell recruitment, activation, and ECM deposition [238]. Neutralizing antibodies or small molecules targeting these proteins have demonstrated efficacy in preclinical models of inflammatory and fibrotic skin diseases [239]. For instance, antibodies against tenascin-C reduce inflammation and fibrosis in experimental models of scleroderma, while osteopontin blockade attenuates psoriasiform inflammation [240,241].

Future therapeutic strategies could focus on developing humanized monoclonal antibodies or small molecule inhibitors targeting these proteins, building upon preclinical successes such as tenascin-C blockade in scleroderma models and osteopontin inhibition in psoriasiform inflammation. Combination approaches that simultaneously target multiple matricellular proteins may offer synergistic benefits, while biomarker-driven patient stratification could identify individuals most likely to respond to specific matricellular protein-targeted therapies. Additionally, developing reversible inhibitors with favorable pharmacokinetic profiles and exploring topical delivery systems could enhance therapeutic efficacy while minimizing systemic side effects, ultimately translating these promising preclinical findings into effective clinical interventions for conditions like scleroderma, psoriasis, and other inflammatory dermatoses.

### 7.5. Matrikine-Based Therapeutics

Synthetic matrikines or matrikine antagonists could modulate inflammation with potentially fewer side effects than systemic immunosuppression. Both pro-regenerative matrikines (promoting M2 polarization and tissue repair) and anti-inflammatory matrikines (neutralizing DAMP signaling) deserve clinical investigation [242]. Engineered hybrid molecules combining matrikine sequences with targeting moieties could achieve localized immunomodulation.

Hyaluronan oligosaccharides of specific molecular weights have demonstrated the ability to modulate macrophage polarization, reduce neutrophil recruitment, and promote tissue repair in experimental models of skin inflammation and wound healing [243]. The small leucine-rich proteoglycan decorin exerts anti-inflammatory and anti-fibrotic effects by sequestering TGF-β, modulating collagen fibrillogenesis, and regulating immune cell behavior [244]. Recombinant decorin and decorin-derived peptides are being explored as therapeutics for fibrotic skin diseases and tumor immunotherapy enhancement [244].

DAMPs derived from matrikines during tissue injury or inflammation constitute critical initiators and amplifiers of immune responses. Low-molecular-weight hyaluronan fragments, fibronectin fragments containing extra domain A (EDA-FN), and versican fragments all signal through pattern recognition receptors such as TLR2, TLR4, and RAGE to activate innate immunity [12,18,245]. Therapeutic strategies to neutralize these ECM-derived DAMPs include antibodies targeting specific fragments, decoy receptors, or inhibitors of the proteases that generate immunostimulatory fragments [110]. Such approaches have shown promise in attenuating chronic inflammation in preclinical models of wound healing disorders and autoimmune skin diseases.

Other ECM-derived peptides with immunomodulatory activity include matrikines generated from collagen (such as Pro-Gly-Pro tripeptides), elastin (elastin-derived peptides), and laminin (laminin-derived sequences like YIGSR and IKVAV) [242,246,247]. While most therapeutic development has focused on wound healing and tissue regeneration, the immunomodulatory properties of these bioactive fragments suggest potential applications in inflammatory dermatoses.

Future matrikine-based therapeutics hold promise for treating inflammatory disorders through targeted immunomodulation with reduced systemic side effects. Clinical development should prioritize synthetic matrikines or antagonists that either promote tissue repair via M2 macrophage polarization or block DAMP-mediated inflammation through pattern recognition receptor inhibition. Engineered hybrid molecules combining matrikine sequences with tissue-targeting moieties could enable localized delivery, while specific molecular weight hyaluronan oligosaccharides and decorin-derived peptides show potential for modulating fibrotic diseases and chronic inflammation. Therapeutic strategies neutralizing ECM-derived DAMPs, such as antibodies against hyaluronan fragments, fibronectin EDA domains, or versican fragments, along with protease inhibitors preventing immunostimulatory fragment generation, warrant investigation in inflammatory dermatoses and wound healing disorders. Additionally, bioactive peptides from collagen, elastin, and laminin merit translation from regenerative medicine to immunomodulatory applications in skin disease, particularly given their demonstrated ability to regulate innate immunity while promoting physiologic tissue repair.

### 7.6. Targeting YAP/TAZ Mechanosensitive Signaling Pathway

The mechanical properties of the ECM have emerged as powerful regulators of inflammation through mechanotransduction pathways, particularly involving YAP/TAZ signaling. Therapeutic strategies such as restoring dermal mechanical properties through ECM scaffolds or crosslinking agents, directly enhancing YAP/TAZ activity through mechanical or biochemical means, or pharmacologically inhibiting STING signaling represent promising approaches for combating age-related skin dysfunction and reversing the inflammatory milieu that perpetuates tissue deterioration.

Future interventions could focus on biomaterial scaffolds engineered to restore optimal tissue stiffness and ECM architecture, thereby reactivating mechanotransduction signaling in aged skin dermis. Small molecule activators of YAP/TAZ or inhibitors of their negative regulators (such as LATS1/2 kinases) may enhance fibroblast function and tissue repair capacity. Additionally, combination approaches integrating mechanical restoration through crosslinking agents or topical formulations that modulate substrate stiffness alongside pharmacological STING pathway inhibition could synergistically reduce chronic inflammation while promoting tissue regeneration.

### 7.7. Elimination of Senescent Fibroblasts

Elimination of senescent fibroblasts through senolytic agents (compounds that selectively induce apoptosis in senescent cells) or suppression of the SASP through senomorphic compounds (which inhibit SASP secretion without killing senescent cells) represents a promising therapeutic strategy for mitigating chronic inflammation and age-related skin disorders [248,249]. Preclinical studies have demonstrated that senolytic combinations such as dasatinib plus quercetin, or novel agents like fisetin and navitoclax, can effectively clear senescent cells and improve tissue function in aged skin models [250]. Similarly, senomorphic approaches targeting NF-κB signaling, mTOR, or JAK/STAT pathways have shown efficacy in suppressing SASP production [251]. Topical and systemic delivery of these agents is under investigation for treating photoaging, chronic wounds, and inflammatory dermatoses, with early clinical trials showing encouraging results in improving skin appearance, reducing inflammatory markers, and enhancing wound healing capacity in aged individuals [251,252].

Based on these findings, future therapeutic strategies should focus on developing targeted senolytic and senomorphic interventions for age-related skin conditions. Priority areas include optimizing topical and systemic delivery systems for compounds like dasatinib–quercetin combinations, fisetin, and navitoclax to enhance skin penetration and bioavailability while minimizing systemic side effects. Additionally, developing biomarkers to monitor senescent cell burden and SASP activity in skin tissue would enable personalized treatment approaches and real-time assessment of therapeutic efficacy. Combination strategies that integrate senolytics with senomorphics targeting specific pathways (NF-κB, mTOR, JAK/STAT) may offer synergistic benefits, warranting investigation in both preclinical models and clinical settings to maximize anti-inflammatory effects while preserving tissue regenerative capacity.

## 8. Future Directions

The matrisome is far more than the sum of ECM and ECM-associated proteins; it acts as a critical interface between tissue structure and immune function, translating environmental changes into inflammatory responses and enabling inflammation to reshape tissue architecture. Unlocking these intricate relationships holds transformative potential for addressing inflammatory diseases (e.g., rheumatoid arthritis, asthma), mitigating fibrosis (such as in pulmonary or hepatic fibrosis), improving cancer therapies (by normalizing tumor microenvironments), managing aging-related disorders (where ECM alterations contribute to immunosenescence and inflammaging), and advancing regenerative medicine. The coming years promise exciting discoveries as technological advances enable unprecedented resolution of matrisome–immune interactions, mechanistic insights drive therapeutic innovation, and clinical translation brings hope to patients suffering from conditions rooted in dysregulated matrisome-inflammation crosstalk. The future of immunology and tissue biology lies in appreciating the matrisome not as a passive backdrop, but as an active protagonist in the drama of inflammation and disease.

### 8.1. High-Resolution Mapping of Matrisome–Immune Interactions

Future research must leverage advanced technologies to achieve spatiotemporal resolution of matrisome–immune cell interactions at the molecular level. Single-cell RNA sequencing combined with spatial transcriptomics can reveal how individual immune cell subsets respond to specific matrisome components within distinct tissue microenvironments. Multiplexed imaging technologies enabling simultaneous visualization of dozens of matrisome proteins, immune markers, and signaling molecules will provide unprecedented insight into the architectural organization of inflammatory niches. Mass spectrometry-based proteomics can comprehensively catalog matrikine species generated during different phases of inflammation, identifying novel bioactive fragments and their receptor targets. Such high-resolution mapping will enable identification of tissue-specific and disease-specific matrisome signatures that could serve as diagnostic biomarkers and therapeutic targets.

### 8.2. Mechanobiology of Inflammation

The mechanical regulation of inflammation through matrisome properties requires deeper investigation. Key questions include: how do specific changes in matrix stiffness, viscoelasticity, and topography influence different immune cell subsets? What are the precise molecular mechanisms linking mechanical sensing through integrins, YAP/TAZ, Piezo channels, and other mechanosensors to inflammatory gene expression programs? How do aging-related changes in dermal mechanical properties contribute to immunosenescence and inflammaging? Can therapeutic restoration of tissue mechanical properties reverse pathological inflammation? Advanced biomaterials with tunable mechanical properties, combined with real-time imaging of mechanotransduction events and genetic manipulation of mechanosensitive pathways, will be essential for addressing these questions. Understanding mechanobiology could reveal entirely new classes of anti-inflammatory interventions based on manipulating tissue physical properties rather than blocking specific molecular targets.

### 8.3. Matrikine Signaling Networks

The signaling mechanisms and receptor repertoires for matrikines remain incompletely characterized. Many matrikines likely act through multiple receptors and exhibit context-dependent activities that vary with concentration, co-presentation with other signals, and cellular phenotype. Future research should systematically identify receptors for known matrikines, elucidate downstream signaling cascades, and determine how matrikine signals integrate with cytokine and growth factor pathways. Structure-function studies can identify minimal bioactive sequences within matrikines, enabling development of synthetic peptides or peptidomimetics as therapeutic agents. Understanding whether specific matrikines exhibit tissue-specific or cell-type-specific activities could enable targeted immunomodulation. Additionally, investigating how post-translational modifications of matrikines (glycosylation, sulfation, phosphorylation) alter their biological activities represents an unexplored frontier.

## 9. Conclusions

The matrisome has emerged as a dynamic and multifaceted regulator of inflammation, fundamentally reshaping our understanding of tissue immunity and inflammatory disease pathogenesis. Far from serving merely as a passive structural scaffold, the matrisome actively orchestrates immune responses through diverse mechanisms: integrin–mediated cellular interactions that guide immune cell trafficking and activation, mechanical signaling that translates tissue stiffness into inflammatory programs, bioactive matrikines that function as damage signals and immunomodulators, and sequestration of cytokines and growth factors that create localized inflammatory microenvironments.

The bidirectional relationship between matrisome and inflammation represents a critical paradigm in modern immunology. Inflammatory mediators drive extensive matrisome remodeling through altered synthesis, increased proteolytic degradation, and chemical modification of ECM components. Reciprocally, this remodeled matrisome perpetuates and amplifies inflammatory responses by releasing pro-inflammatory fragments, exposing cryptic epitopes, altering mechanical properties that activate mechanosensitive pathways, and creating tissue microenvironments that sustain pathological immune cell phenotypes. This creates self-reinforcing cycles that underline chronic inflammatory diseases, fibrosis, impaired wound healing, and aging-related tissue dysfunction.

Understanding the immunomodulatory roles of matrisome provides important insights into numerous therapeutic opportunities. Targeting matrisome, modulating matrisome–immune interactions, inhibiting pathological ECM remodeling, and manipulating ECM properties represent promising strategies for treating inflammatory diseases. As technologies advance and our understanding deepens, matrisome-directed therapies may achieve more precise control of tissue inflammation with improved efficacy and safety profiles. The integration of advanced technologies including matrisome proteomics combine with single-cell omics, and spatial transcriptomics will accelerate progress toward these goals. Ultimately, recognizing matrisome as dynamic immunomodulators rather than passive structural protein fundamentally reshapes our understanding of inflammation and offers hope for improved therapeutic strategies.

## Data Availability

No new data were created or analyzed in this study.
